# Supplementary material for: Causal Relationship between Aging and Anorexia Nervosa: A White-Matter-Microstructure-Mediated Mendelian Randomization Analysis
Source: Biomedicines. 2024 Aug 16;12(8):1874. doi: 10.3390/biomedicines12081874 (PMC11351342; doi:10.3390/biomedicines12081874)
Supplement: Supplementary file 1 [file biomedicines-12-01874-s001.zip › Supplementary Figure/Figure S8/Leaveoneout plot of MR analysis for TL to ubm-b-2092.pdf]

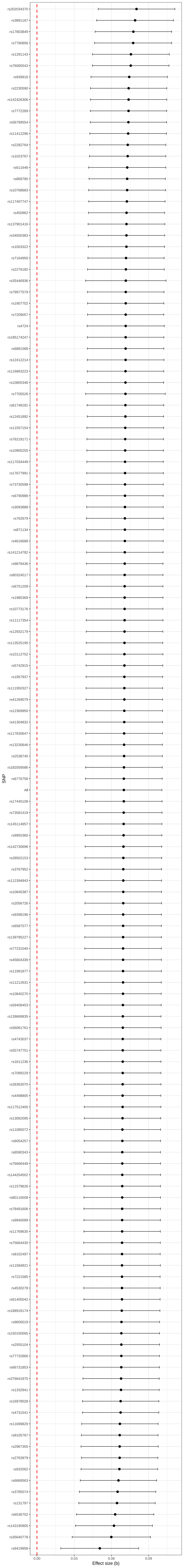

rs202034370
rs3891167
rs17803849
rs7790856
rs1291143
rs76065543
rs939916
rs2230590
rs142426306
rs7772289
rs56799554
rs11412296
rs2282764
rs1023767
rs611646
rs869785
rs10768683
rs117407747
rs450962
rs137901416
rs34550383
rs1003322
rs7164950
rs2276182
rs35446936
rs79977579
rs1907702
rs7209057
rs4724
rs185174247
rs6881568
rs12412214
rs116863223
rs10805346
rs7705526
rs61748181
rs12451892
rs11557154
rs76219171
rs10905255
rs117034449
rs17677991
rs73730598
rs6790988
rs3093888
rs762679
rs871134
rs4616688
rs141214782
rs9878436
rs80324517
rs6751209
rs1985369
rs10773176
rs11117354
rs12932170
rs113525195
rs10112752
rs5742915
rs1957937
rs111950327
rs41269079
rs12369950
rs41304832
rs117630647
rs13230646
rs2538745
rs182059586
rs6776756
A8
rs17445108
rs73581419
rs145114957
rs9955360
rs142730696
rs28502153
rs3767952
rs112394943
rs10645387
rs2056726
rs9398196
rs6587577
rs139795227
rs77231040
rs45604339
rs11991877
rs11212631
rs10840270
rs59409453
rs139669835
rs56061761
rs4743037
rs55747751
rs1611236
rs7099229
rs28363070
rs4498805
rs117512405
rs13062095
rs11085072
rs6054257
rs6590343
rs76666449
rs144204502
rs11579626
rs80116508
rs78491606
rs9940099
rs11769630
rs75664430
rs8102497
rs11584821
rs7221585
rs4530278
rs61405042
rs188918174
rs9600019
rs150150565
rs2555104
rs77732866
rs66731853
rs376641875
rs1332941
rs16978028
rs4731541
rs11699829
rs8105767
rs2967355
rs2763979
rs932002
rs6669563
rs3785074
rs131797
rs6536702
rs143190905
rs35640778
rs9419958
SNP
0.00
0.03
0.06
0.09
Effect size (b)
